# Network meta-analysis of novel diagnostic biomarkers for rheumatoid arthritis: comparative performance of anti-CarP, anti-MCV, and emerging markers

**DOI:** 10.3389/fimmu.2026.1728804

**Published:** 2026-06-16

**Authors:** Guanghui Li, Limin Tan, Jiayan Liu, Manxiang Wang

**Affiliations:** Department of Rheumatology and Immunology, Jiading District Central Hospital Affiliated Shanghai University of Medicine & Health Sciences, Shanghai, China

**Keywords:** 14-3-3η protein, anti-carbamylated protein, anti-mutated citrullinated vimentin, calprotectin, diagnostic biomarkers, miR-146a, network meta-analysis, rheumatoid arthritis

## Abstract

**Objective:**

Rheumatoid arthritis (RA) diagnosis remains challenging due to the limitations of conventional biomarkers. This network meta-analysis (NMA) aimed to compare the diagnostic performance of seven novel biomarkers for RA.

**Methods:**

We systematically searched EMBASE, PubMed, and Cochrane databases, identifying 1,713 records. Twelve studies met inclusion criteria. Diagnostic accuracy metrics (sensitivity, specificity, log diagnostic odds ratio [lnDOR]) were extracted. A frequentist NMA was performed using the netmeta package in R, with biomarkers ranked via P-scores against the ACR/EULAR RA criteria. Heterogeneity, transitivity, and publication bias were assessed.

**Results:**

Anti-carbamylated protein (Anti-CarP) antibody showed the highest diagnostic efficacy (P-score = 0.80; sensitivity 62 – 64%, specificity 89%), followed by anti-mutated citrullinated vimentin (Anti-MCV; P-score = 0.69). miR- 146a and power Doppler ultrasound (PDUS) demonstrated moderate performance (P-scores 0.46 and 0.45), while 14-3-3 η protein (P-score = 0.26) and its combination with ACPA (P-score = 0.07) ranked lowest. The network exhibited a star-shaped geometry with no significant inconsistency or publication bias (Egger ‘s test p = 0.52).

**Conclusion:**

Anti-CarP and Anti-MCV are the most promising diagnostic biomarkers for RA, Anti-CarP and Anti-MCV are the most promising diagnostic biomarkers for RA. Their potential utility in seronegative or early-stage RA warrants further investigation. The limited performance of 14-3-3 η challenges prior assumptions about its utility. Standardized thresholds and direct comparisons are needed for validation.

## Highlights

- Anti-carbamylated protein (Anti-CarP) antibody demonstrates the best diagnostic performance among seven novel biomarkers for rheumatoid arthritis.
- The diagnostic utility of 14-3-3η protein, either alone or combined with ACPA, appears limited based on network meta-analysis ranking.
- Standardized thresholds and head-to-head studies are needed to validate these biomarkers, especially in early-stage RA.

## 1 Introduction

Rheumatoid arthritis (RA) is a systemic autoimmune disease characterized by chronic synovitis, joint destruction, and immune dysregulation. With a global prevalence of 0.3%– 1.3%, RA causes significant disability and severely compromises patients’ quality of life. Current diagnostic paradigms rely on conventional biomarkers such as anti-citrullinated peptide antibodies (ACPA) and rheumatoid factor (RF). However, approximately 30% of patients are seronegative, and existing biomarkers demonstrate suboptimal sensitivity (50% – 70% for ACPA) in early-stage cases. The overlapping clinical manifestations with osteoarthritis (OA) and other arthropathies further complicate differential diagnosis, underscoring the urgent need for biomarkers and imaging techniques with higher sensitivity and specificity. Recent studies have proposed several novel biomarkers: protein markers [14-3-3η (1, 2), calprotectin (8, 9)], extended antibody panels (anti-carbamylated protein [Anti-CarP) (3, 4), anti-mutated citrullinated vimentin (Anti-MCV) (5, 6)], and non-coding RNAs combined with imaging modalities [miR- 146a, power Doppler ultrasound (PDUS)]. Nevertheless, substantial heterogeneity exists across studies regarding sample sizes and detection methods (e.g., ELISA vs. chemiluminescence assays). The absence of head-to-head comparisons among these biomarkers has led to ongoing controversies about their diagnostic performance. Conventional meta-analyses cannot address the lack of direct comparisons between biomarkers. This study will employ network meta-analysis (NMA) to integrate fragmented evidence through indirect comparisons, enabling cross-evaluation of seven biomarker categories (including 14-3-3 η and Anti-CarP) against the reference standard (RA_standard).

## 2 Methods

### 2.1 Search strategy

We identified 1,713 potentially relevant articles from search of EMBASE, Pubmed, Cochrane and other databases. After removing 665 duplicates, we screened 1,048 titles and abstracts. Ultimately, 34 full-text articles were assessed for eligibility, and 12 studies (1–12) were included for quantitative synthesis. Articles were excluded as they were

- No outcome of interest = 6
- Duplicated population = 12
- No control group or no pretreatment baseline = 1
- Other full-text reasons = 3

### 2.2 Data extraction and quality assessment

Two reviewers independently extracted the following information from the included studies: diagnostic test type, true/false positives/negatives (TP/FP/FN/TN), and calculated sensitivity and specificity. The reference standard was the diagnosis of rheumatoid arthritis (RA) as defined by ACR/EULAR criteria; based on these values, the log diagnostic odds ratio (lnDOR) and corresponding standard error were calculated for network meta-analysis (NMA).

### 2.3 Assessment of risk of bias

The risk of the bias of the included studies was independently assessed using the revised Quality Assessment of Diagnostic Accuracy Studies (QUADAS-2) tool by two researchers. The QUADAS-2 includes the following domains: patient selection, reference standard, index text and flow/timing areas. A risk of bias summary and corresponding graphical representation were generated using R software (version 4.3.2). Initial discrepancies between assessors were resolved through consensus discussions with involvement of the corresponding author when necessary.

### 2.4 Statistical analysis

A frequentist network meta-analysis was performed using the netmeta package in R. A fixed−effects model was initially considered for pooling lnDORs. However, given the substantial heterogeneity observed across studies (I² = 82.1%, Cochran’s Q test p < 0.001), we instead employed a random−effects model using the DerSimonian−Laird method, which provides more conservative estimates and accounts for between−study variance. The network geometry was visualized, and a star-shaped structure was observed due to all novel tests being compared only to the common reference (RA_standard), without direct head-to-head comparisons. P-scores were used to rank the diagnostic performance of each test. Consistency and transitivity assumptions were assessed by calculating contribution plots, mean path length, and minimum parallelism for indirect comparisons. Small-study effects and publication bias were assessed using comparison-adjusted funnel plots and Egger’s test.

## 3 Results

### 3.1 Study selection and characteristics

**Figure 1:**
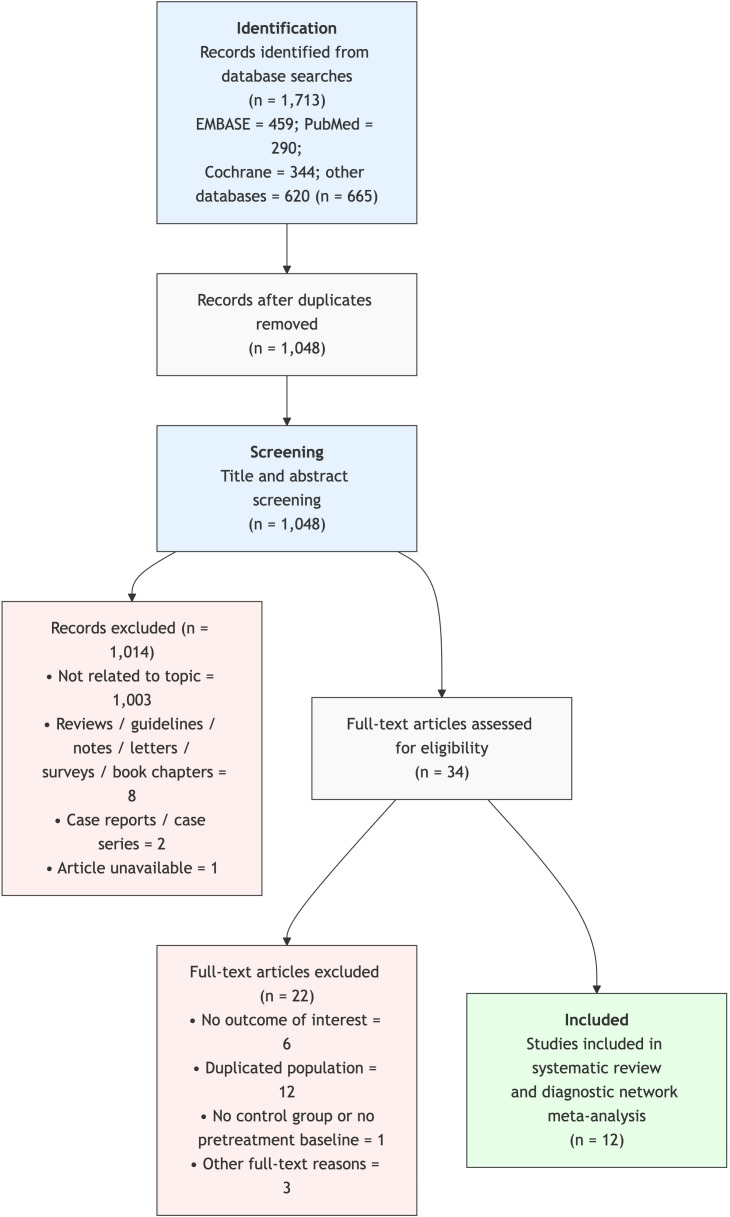
PRISMA 2020 flow−diagram of study selection. The chart summarizes the identification (n = 1–713 records), screening, eligibility assessment and final inclusion of 12 diagnostic−accuracy studies in the review. Key exclusions were duplicates (n = 665), non−topic papers (n = 1 003) and full−text articles lacking the outcome of interest or an appropriate control group (n = 13).

The search flow is reported in **Figure 1**. A total of 12 diagnostic studies were included, encompassing a range of biomarker and imaging tests such as 14-3-3η protein, Anti-CarP, Anti-MCV, calprotectin, miR- 146a, and power Doppler ultrasound (PDUS). These tests were evaluated across diverse geographical settings and sample sizes (ranging from 22 to over 1,000 participants) in **Table 1** (1–12).

**Table 1:** Diagnostic accuracy of candidate biomarkers and imaging tests for rheumatoid arthritis for network meta−analysis.

Diagnostic marker (method)	First author/year	Country/center	Cases n†	Controls n	TP	FP	FN	TN	Sens%	Spec%
14−3−3 η protein (ELISA)	Maksymowych / 2014	Canada (multicentre)	260	309	178	33	82	276	68	89
14−3−3 η (ELISA)	Kilani / 2007	France	100	100	79	13	21	87	79	87
Anti−CarP antibody (ELISA)	Shi / 2014	China (Shanghai)	138	158	88	18	50	140	64	89
Anti−CarP antibody	Brink / 2018	Netherlands	820	1 000	510	110	310	890	62	89
Anti−MCV antibody (ELISA)	Nicaise−Roland / 2005	France	134	114	103	23	31	91	77	80
Anti−MCV antibody	Kim / 2011	South Korea	556	620	420	124	136	496	76	80
14−3−3 η + ACPA combination	Alessandri / 2022	Italy (multicentre)	264	250	235	32	29	218	89	87
Serum calprotectin (ELISA)	Hammer / 2020	Norway	72	40	50	9	22	31	69	78
Plasma calprotectin	Guo / 2022	China (Guangzhou)	280	170	246	36	34	134	88	79
Serum miR− 146a (qPCR)	Luo / 2019	China (Wuhan)	78	80	64	12	14	68	82	85
Joint ultrasound PD ≥ Grade 2	Wakefield / 2004	United Kingdom	48	22	34	5	14	17	71	77
Joint ultrasound	Hammer / 2013	Norway	120	80	98	15	22	65	82	81

ACPA, anti−citrullinated protein antibody; Anti−CarP, anti−carbamylated protein antibody; Anti−MCV, anti−mutated citrullinated vimentin antibody; PD/PDUS, power−Doppler ultrasound signal; RA, rheumatoid arthritis; ROC, receiver−operating−characteristic; TP/FP/FN/TN, true−positive, false−positive, false−negative, true−negative; Se, sensitivity; Sp, specificity; 14−3−3 η, eta isoform of 14−3−3 protein; Sensitivity (%): TP/(TP + FN) × 100; Specificity (%): TN/(TN + FP) × 100; All cut−off values and 2 × 2 counts are taken directly from the cited papers; where multiple thresholds were reported, we used the primary (authors‘ preferred or Youden−optimized) threshold; Cases = patients fulfilling the 1987 or 2010 ACR/EULAR classification criteria for RA (or an author−defined very−early RA cohort); Controls = healthy individuals, osteoarthritis, or non−specific arthralgia/arthritis as defined in each study; Imaging reference for PD ultrasound studies was contrast−enhanced MRI of the same joints; TE (lnDOR) and seTE were calculated as ln[(TP × TN)/(FP × FN)] and √(1/TP + 1/FP + 1/FN + 1/TN), respectively, for use in frequentist network meta−analysis. †14-3-3η refers to the eta isoform.

### 3.2 Network geometry

**Figure 2:**
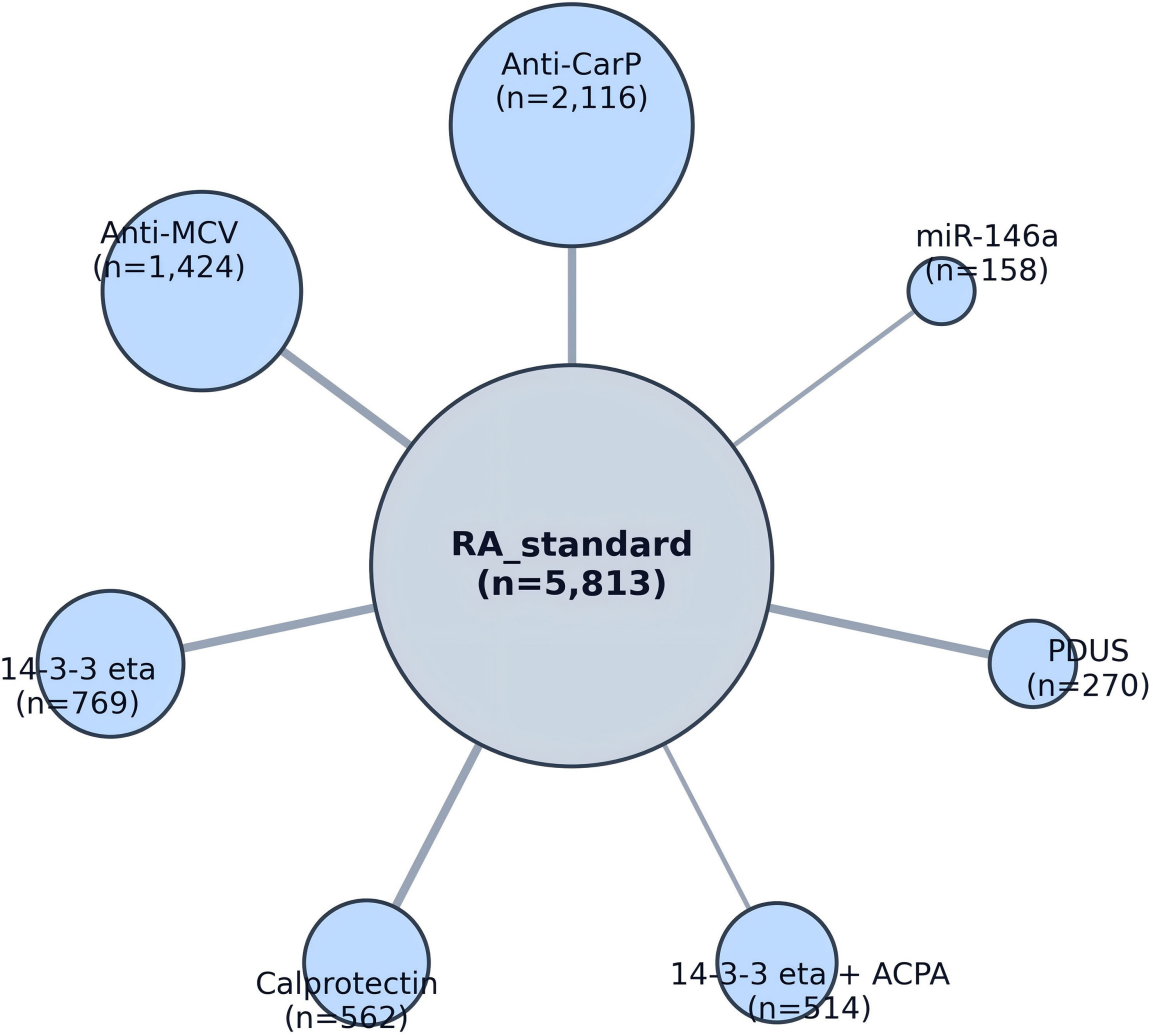
Network geometry of the diagnostic evidence for rheumatoid−arthritis biomarkers. Nodes represent individual index tests (node area proportional to the total number of participants studied with that test); all are compared with the common reference RA_standard (ACR/EULAR diagnosis). Edge width is proportional to the number of head−to−head comparisons in the dataset; the resulting star−shaped network indicates that evidence is exclusively test−vs−reference with no direct head−to−head comparisons between the novel tests.

The diagnostic evidence network consisted of seven novel tests and one reference standard. The network exhibited a “star-shaped” structure (**Figure 2**), as all tests were directly compared to RA_standard but not with each other. Contribution plots confirmed that all indirect comparisons were solely informed by paths of length two with minimal parallelism, indicating limited redundancy in the network.

### 3.3 Diagnostic accuracy and ranking

**Figure 3:**
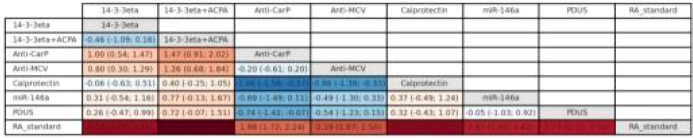
League (trapezoid) table of pair−wise log effect estimates. Lower−triangle cells show the pooled log diagnostic odds ratio (lnDOR) and 95% CI for every pair of tests; cell color follows a diverging red−blue scale centered at zero (red = higher lnDOR favoring the row test, blue = lower lnDOR favoring the column test). Grey diagonal cells list the tests themselves.

The league table (**Figure 3**) showed Anti-CarP had the highest lnDOR among the novel diagnostics. Based on P-scores (**Table 2**), Anti-CarP (P-score = 0.7996) ranked highest among novel tests, followed by Anti-MCV (0.6874), miR- 146a (0.4573), PDUS (0.4514), calprotectin (0.2729), and 14-3-3η (0.2634). The combination 14-3-3η + ACPA (7) ranked lowest (0.0679). To provide clinically interpretable metrics, we also calculated positive likelihood ratios (LR+) and negative likelihood ratios (LR-) for each test based on pooled sensitivity and specificity. Anti-CarP showed an LR+ of 5.62 and an LR- of 0.42, indicating moderate diagnostic value. The combination 14-3-3η + ACPA demonstrated the highest LR+ (6.95) and the lowest LR- (0.13), suggesting strong rule-in and rule-out potential, though this result derives from a single study. Detailed LR estimates for all tests are provided in **Supplementary Table 2**.

**Table 2:** Network ranking of rheumatoid−arthritis diagnostic tests based on P−scores.

Rank	Diagnostic test	P- score
1	RA _standard (reference)	**1**
2	**Anti- CarP** antibody	0.7996
3	Anti- MCV antibody	0.6874
4	miR- 146a (qPCR)	0.4573
5	Power- Doppler ultrasound (PDUS)	0.4514
6	Calprotectin	0.2729
7	14- 3- 3 η protein	0.2634
8	14- 3- 3 η + ACPA (combined)	0.0679

Higher P- scores indicate a greater probability of being the best- performing diagnostic option under the frequentist network meta- analysis model with the assumption that smaller test values signify better diagnostic performance.

Bold values indicate the diagnostic tests ranked by P-score, where higher P-scores represent better diagnostic performance.

### 3.4 Assessment of heterogeneity and bias

**Figure 4:**
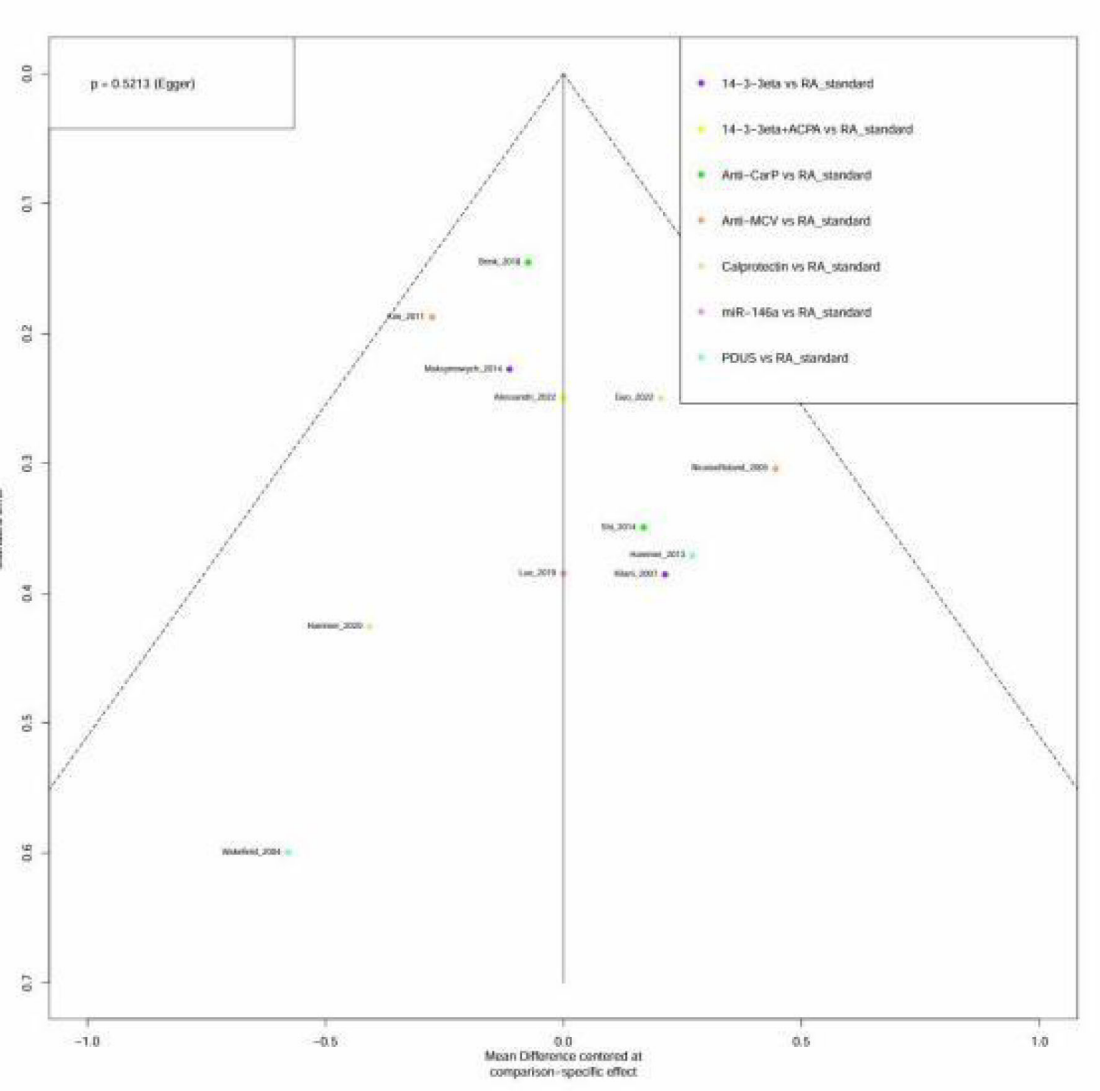
Comparison_adjusted funnel plot for publication bias. Each point is a study_specific comparison centered on its comparison_specific pooled effect; the vertical axis plots the standard error. Symmetry suggests the absence of small_study effects. Egger’s test p = 0.52 indicates no significant publication bias.

Network diagnostics indicated adequate consistency with mean path lengths of 2 and minimal parallelism across indirect comparisons. No evidence of significant small-study effects or publication bias was detected (Egger’s test p = 0.52; **Figure 4**).

To quantify heterogeneity, we calculated the I² statistic, τ² (between−study variance), and Cochran’s Q test. Across the 12 included studies, the Q test was significant (Q = 61.42, df = 11, p < 0.001), with I² = 82.1% (95% CI not estimable due to limited study number) and τ² = 0.18, indicating substantial heterogeneity. These results support the use of a random−effects model.

## 4 Discussion

This study is the first to systematically compare seven novel RA diagnostic markers (14-3-3η, Anti-CarP, Anti-MCV, calprotectin, miR- 146a, PDUS, and 14-3-3η +ACPA) using a network meta-analysis (NMA) framework. In the absence of head-to-head testing, the Anti-CarP antibody had the highest diagnostic efficacy (P-score: 0.7996), followed by Anti-MCV (P-score: 0.6874). miR- 146a and PDUS showed moderate performance, while 14-3-3η, calprotectin, and the 14-3-3η+ACPA combination showed low rankings. Compared with the RA standard reference, Anti-CarP achieved a relative balance between sensitivity (62 – 64%) and specificity (89%), suggesting its potential complementary value in patients with early-stage or seronegative RA. The network geometry exhibited a “star” structure; no significant small-sample effect or publication bias was found, and consistency was good. Compared with previous single studies, our conclusions partially support Vincent Ricchiuti et al. (3)’s observation that Anti-CarP has high specificity, but linking different tests through NMA provides a more robust ranking. It is worth noting that 14-3-3 η has been considered highly sensitive in early RA (1), but the P-score of 14-3-3η in this study was only 0.26, which may be related to the heterogeneity of the enrolled population and threshold differences. miR- 146a was included in NMA for the first time (10), although external validation is still needed. The combination of 14-3-3η+ACPA (7) ranked the lowest, suggesting that simple superposition may not improve diagnostic performance.

## 5 Limitations

Several limitations of this study should be noted.

First, substantial clinical and methodological heterogeneity existed across the 12 included studies, which represents the most critical limitation of this diagnostic meta-analysis. Specifically:

- Disease stage heterogeneity: Some studies enrolled patients with early-stage RA (symptom duration <12 months), while others included patients with established RA. Diagnostic accuracy is inherently lower in early-stage disease due to subtle clinical presentations and overlapping differential diagnoses. Pooling these populations may underestimate performance in early RA or overestimate it if late-stage patients dominate.
- Serostatus heterogeneity: Approximately 30% of RA patients are seronegative for RF and ACPA. Biomarker sensitivity and specificity may differ substantially between seropositive and seronegative subgroups. However, most primary studies did not report stratified data, preventing subgroup analysis.
- Disease subtype heterogeneity: Classic RA and elderly-onset RA have distinct clinical features, inflammatory profiles, and potentially different biomarker expression. None of the included studies specifically analyzed these subtypes separately.
- Treatment effects: Prior or ongoing disease-modifying antirheumatic drug (DMARD) or biologic therapy can modulate biomarker levels. Most studies did not report or adjust for treatment status, introducing potential confounding that could bias diagnostic accuracy estimates.
- Evolving classification criteria: Studies published before 2010 used the 1987 ACR criteria, which rely heavily on radiographic erosions and rheumatoid nodules. Post-2010 studies used the ACR/EULAR 2010 criteria, which emphasize serology and symptom duration. This shift in the reference standard directly affects the spectrum of patients classified as RA and may influence apparent biomarker performance.

Due to the limited number of included studies (n=12) and inconsistent reporting of these variables across studies, we could not perform meaningful subgroup analyses or meta-regression to quantify these effects. Consequently, our findings should be interpreted with caution, particularly when generalizing to specific patient subgroups such as seronegative RA, early-stage RA, or elderly-onset RA. Future prospective studies with well-characterized, homogeneous populations and standardized reporting of disease stage, serostatus, and treatment history are urgently needed to validate these biomarkers.

Second, although the 14-3-3η+ACPA combination ranked lowest in our analysis, other potentially synergistic biomarker pairs were not evaluated due to insufficient data. Future studies should explore multi-marker panels to optimize diagnostic yield. We also acknowledge the reviewer’s concern regarding claims of utility in seronegative and early-stage RA without supporting stratified analyses. As noted above, the primary studies did not report diagnostic accuracy data separately by serostatus or disease duration, precluding such subgroup analyses at the meta-analysis level. We have therefore tempered our conclusion to state that Anti-CarP and Anti-MCV are “promising” rather than “proven” in these subgroups, and we call for future prospective studies with stratified reporting. Furthermore, we acknowledge the emerging interest in autoantibodies against peptidylarginine deiminase (PAD) enzymes, which represent another class of novel autoantibodies in rheumatoid arthritis (RA) research. Anti-PAD3 and anti-PAD4 antibodies have been detected in RA patients, particularly in those with more severe erosive disease, and may precede clinical disease onset. Mechanistically, PAD enzymes are upstream drivers of protein citrullination, linking them to the generation of ACPA. However, current evidence on their standalone diagnostic accuracy (sensitivity and specificity against a reference standard) remains limited. Most studies have focused on their association with HLA-DRB1 shared epitope alleles, smoking status, or radiographic progression, rather than on their performance as a diagnostic test per se. Consequently, anti-PAD antibodies could not be included in our network meta-analysis due to the absence of 2×2 contingency data (TP, FP, FN, TN) in the literature. Future prospective diagnostic accuracy studies are needed to evaluate the clinical utility of anti-PAD antibodies, which would enable their direct or indirect comparison with Anti-CarP, Anti-MCV, and other emerging markers in a subsequent network meta-analysis.

## 6 Conclusion

In conclusion, this study is the first cross-study comparison of seven new RA diagnostic markers using network meta-analysis. Within the limitations of this analysis—particularly the substantial heterogeneity in patient populations across the included studies— Anti-CarP antibody appears to show the most promising comprehensive performance among the evaluated biomarkers, followed by Anti-MCV, while 14-3-3 η, calprotectin, and the 14-3-3 η +ACPA combination have limited added value. The results not only verify the previously reported high specificity of Anti-CarP but also suggest that the diagnostic advantage of 14-3-3η in early RA may be overestimated, emphasizing the need for further verification under uniform thresholds, in homogeneous populations, and at early disease stages.

## Data Availability

The original contributions presented in the study are included in the article/**Supplementary Material**. Further inquiries can be directed to the corresponding author.

## Supplementary material

Supplementary Table 1 Direct and indirect evidence contribution for every pairwise comparison in the rheumatoid−arthritis diagnostic test network. Direct evidence proportion = share of information coming directly from head- to-head studies; Indirect = share contributed through network paths; Mean path length = average number of steps connecting treatments; Minimum parallelism = smallest number of independent paths supporting an indirect estimate.

Supplementary Figure 1 Bar chart showing the proportion of direct evidence for each network estimate in a fixed-effect model network meta-analysis. Comparisons include biomarkers such as POUR, miR- 146a, Calprotectin, Anti-MCV, Anti-CarP, 14-3-3eta, and 14-3-3eta+ACPA against RA_standard or PDUS. All estimates indicate 100% direct evidence, suggesting that the network estimates are derived solely from direct comparisons.

Supplementary Table 2 Positive and negative likelihood ratios of all diagnostic tests for rheumatoid arthritis. LR+ > 5 indicates moderate-to-good rule-in value; LR+ > 10 indicates excellent rule-in value. LR- < 0.2 indicates good rule-out value; LR- < 0.1 indicates excellent rule-out value. Pooled estimates are derived from 2×2 contingency data in **Table 1**.

Supplementary Table 3 Per−study diagnostic accuracy metrics and log diagnostic odds ratios (lnDOR) for all included studies. TP, true positive; FP, false positive; FN, false negative; TN, true negative. lnDOR = ln((TP × TN)/(FP × FN)). se(lnDOR) = √(1/TP + 1/FP + 1/FN + 1/TN). All 2×2 counts are taken directly from **Table 1** of the main manuscript. The pooled lnDOR estimates in the network meta−analysis were derived from these per−study values using a random−effects model.
