# Supplementary material for: Network meta-analysis of novel diagnostic biomarkers for rheumatoid arthritis: comparative performance of anti-CarP, anti-MCV, and emerging markers
Source: Front Immunol. 2026 Jun 16;17:1728804. doi: 10.3389/fimmu.2026.1728804 (PMC13314475; doi:10.3389/fimmu.2026.1728804)
Supplement: Supplementary Table 1 — Direct and indirect evidence contribution for every pairwise comparison in the rheumatoid−arthritis diagnostic test network. Direct evidence proportion = share of information coming directly from head- to-head studies; Indirect = share contributed through network paths; Mean path length = average number of steps connecting treatments; Minimum parallelism = smallest number of independent paths supporting an indirect estimate. [file Table1.docx]

Supplementary Table S1.

Direct and indirect evidence contribution for every pairwise comparison in the rheumatoid‑arthritis diagnostic test network

| Comparison | Direct  evidence  proportion | Indirect  evidence  proportion | Mean  path  length | Minimum  parallelism |
| --- | --- | --- | --- | --- |
| 14‑3‑3η vs RA_standard | 1 | 0 | 1 | 1 |
| 14‑3‑3η + ACPA vs RA_standard | 1 | 0 | 1 | 1 |
| Anti‑CarP vs RA_standard | 1 | 0 | 1 | 1 |
| Anti‑MCV vs RA_standard | 1 | 0 | 1 | 1 |
| Calprotectin vs RA_standard | 1 | 0 | 1 | 1 |
| miR‑ 146a vs RA_standard | 1 | 0 | 1 | 1 |
| PDUS vs RA_standard | 1 | 0 | 1 | 1 |
| 14‑3‑3η vs 14‑3‑3η + ACPA | 0 | 1 | 2 | 1 |
| 14‑3‑3η vs Anti‑CarP | 0 | 1 | 2 | 1 |
| 14‑3‑3η vs Anti‑MCV | 0 | 1 | 2 | 1 |
| 14‑3‑3η vs Calprotectin | 0 | 1 | 2 | 1 |
| 14‑3‑3η vs miR‑ 146a | 0 | 1 | 2 | 1 |
| 14‑3‑3η vs PDUS | 0 | 1 | 2 | 1 |

| 14‑3‑3η + ACPA vs Anti‑CarP | 0 | 1 | 2 | 1 |
| --- | --- | --- | --- | --- |
| 14‑3‑3η + ACPA vs Anti‑MCV | 0 | 1 | 2 | 1 |
| 14‑3‑3η + ACPA vs Calprotectin | 0 | 1 | 2 | 1 |
| 14‑3‑3η + ACPA vs miR‑ 146a | 0 | 1 | 2 | 1 |
| 14‑3‑3η + ACPA vs PDUS | 0 | 1 | 2 | 1 |
| Anti‑CarP vs Anti‑MCV | 0 | 1 | 2 | 1 |
| Anti‑CarP vs Calprotectin | 0 | 1 | 2 | 1 |
| Anti‑CarP vs miR‑ 146a | 0 | 1 | 2 | 1 |
| Anti‑CarP vs PDUS | 0 | 1 | 2 | 1 |
| Anti‑MCV vs Calprotectin | 0 | 1 | 2 | 1 |
| Anti‑MCV vs miR‑ 146a | 0 | 1 | 2 | 1 |
| Anti‑MCV vs PDUS | 0 | 1 | 2 | 1 |
| Calprotectin vs miR‑ 146a | 0 | 1 | 2 | 1 |
| Calprotectin vs PDUS | 0 | 1 | 2 | 1 |
| miR‑ 146a vs PDUS | 0 | 1 | 2 | 1 |

Note: Direct evidence proportion = share of information coming directly from

head- to-head studies; Indirect = share contributed through network paths; Mean path length = average number of steps connecting treatments; Minimum

parallelism = smallest number of independent paths supporting an indirect estimate.
