# Supplementary material for: Network meta-analysis of novel diagnostic biomarkers for rheumatoid arthritis: comparative performance of anti-CarP, anti-MCV, and emerging markers
Source: Front Immunol. 2026 Jun 16;17:1728804. doi: 10.3389/fimmu.2026.1728804 (PMC13314475; doi:10.3389/fimmu.2026.1728804)
Supplement: Supplementary Figure 1 — Bar chart showing the proportion of direct evidence for each network estimate in a fixed-effect model network meta-analysis. Comparisons include biomarkers such as POUR, miR- 146a, Calprotectin, Anti-MCV, Anti-CarP, 14-3-3eta, and 14-3-3eta+ACPA against RA_standard or PDUS. All estimates indicate 100% direct evidence, suggesting that the network estimates are derived solely from direct comparisons. [file Supplementaryfile1.docx]

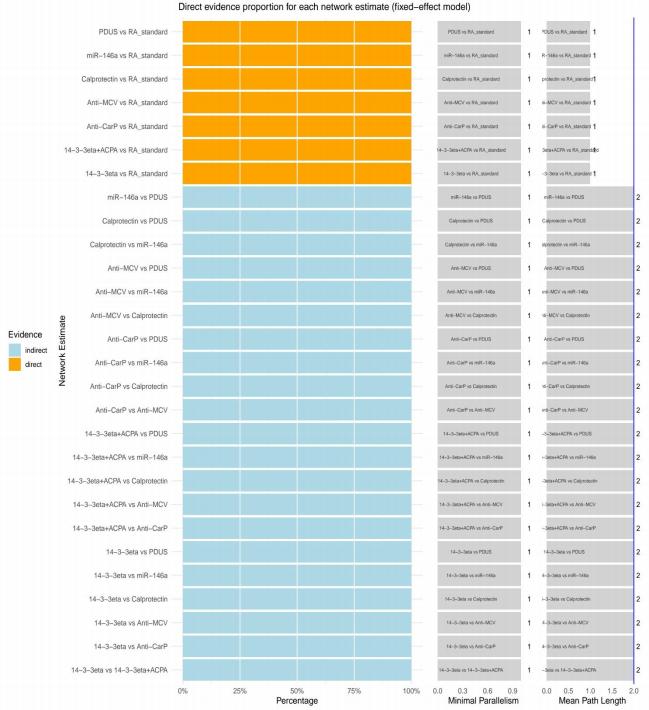


In review

Supplementary Figure S1

ALT TEXT: Bar chart showing the proportion of direct evidence for each network

estimate in a fixed-effect model network meta-analysis. Comparisons include

biomarkers such as POUR, miR- 146a, Calprotectin, Anti-MCV, Anti-CarP, 14-3-3eta, and 14-3-3eta+ACPA against RA_standard or PDUS. All estimates indicate 100%

direct evidence, suggesting that the network estimates are derived solely from direct comparisons.
