# Supplementary material for: Network meta-analysis of novel diagnostic biomarkers for rheumatoid arthritis: comparative performance of anti-CarP, anti-MCV, and emerging markers
Source: Front Immunol. 2026 Jun 16;17:1728804. doi: 10.3389/fimmu.2026.1728804 (PMC13314475; doi:10.3389/fimmu.2026.1728804)
Supplement: Supplementary Table 2 — Positive and negative likelihood ratios of all diagnostic tests for rheumatoid arthritis. LR+ > 5 indicates moderate-to-good rule-in value; LR+ > 10 indicates excellent rule-in value. LR- < 0.2 indicates good rule-out value; LR- < 0.1 indicates excellent rule-out value. Pooled estimates are derived from 2×2 contingency data in Table 1. [file Table2.docx]

Supplementary Table S2.

Positive and negative likelihood ratios of all diagnostic tests for rheumatoid arthritis

| Diagnostic | test LR+ LR- Interpretation |  |  |  |  |  |
| --- | --- | --- | --- | --- | --- | --- |
| 14-3-3η | + | ACPA | (combined) 6.95 0.13 Good | rule-in | and | rule-out |
| 14-3-3η 6.37 0.32 Good | rule-in, | moderate | rule-out |  |  |  |
| Anti-CarP 5.62 0.42 Moderate | rule-in, | fair | rule-out |  |  |  |
| miR-146a 5.47 0.21 Moderate | rule-in, | good | rule-out |  |  |  |
| PDUS 4.01 0.27 Moderate | rule-in, | good | rule-out |  |  |  |
| Calprotectin 3.93 0.20 Moderate | rule-in, | good | rule-out |  |  |  |
| Anti-MCV 3.79 0.30 Moderate | rule-in, | moderate | rule-out |  |  |  |

Note: LR+ > 5 indicates moderate-to-good rule-in value; LR+ > 10 indicates excellent rule-in value. LR- < 0.2 indicates good rule-out value; LR- < 0.1 indicates excellent rule-out value. Pooled estimates are derived from 2×2 contingency data in Table 1.
