# Supplementary material for: Network meta-analysis of novel diagnostic biomarkers for rheumatoid arthritis: comparative performance of anti-CarP, anti-MCV, and emerging markers
Source: Front Immunol. 2026 Jun 16;17:1728804. doi: 10.3389/fimmu.2026.1728804 (PMC13314475; doi:10.3389/fimmu.2026.1728804)
Supplement: Supplementary Table 3 — Per−study diagnostic accuracy metrics and log diagnostic odds ratios (lnDOR) for all included studies. TP, true positive; FP, false positive; FN, false negative; TN, true negative. lnDOR = ln((TP × TN)/(FP × FN)). se(lnDOR) = √(1/TP + 1/FP + 1/FN + 1/TN). All 2×2 counts are taken directly from Table 1 of the main manuscript. The pooled lnDOR estimates in the network meta−analysis were derived from these per−study values using a random−effects model. [file Table3.docx]

Supplementary Table S3.

Per‑study diagnostic accuracy metrics and log diagnostic odds ratios (lnDOR) for all included studies

| Study (first author, year) | Biomarker | TP | FP | FN | TN | lnDOR | se(lnDOR) |
| --- | --- | --- | --- | --- | --- | --- | --- |
| Maksymowych 2014 | 14‑3‑3η protein | 178 | 33 | 82 | 276 | 2.90 | 0.23 |
| Kilani 2007 | 14‑3‑3η protein | 79 | 13 | 21 | 87 | 3.23 | 0.39 |
| Shi 2014 | Anti‑CarP antibody | 88 | 18 | 50 | 140 | 2.62 | 0.31 |
| Brink 2018 | Anti‑CarP antibody | 510 | 110 | 310 | 890 | 2.59 | 0.12 |
| Nicaise‑Roland 2005 | Anti‑MCV antibody | 103 | 23 | 31 | 91 | 2.58 | 0.31 |
| Kim 2011 | Anti‑MCV antibody | 420 | 124 | 136 | 496 | 2.51 | 0.14 |
| Alessandri 2022 | 14‑3‑3η + ACPA | 235 | 32 | 29 | 218 | 4.01 | 0.27 |
| Hammer 2020 | Serum calprotectin | 50 | 9 | 22 | 31 | 2.06 | 0.46 |
| Guo 2022 | Plasma calprotectin | 246 | 36 | 34 | 134 | 3.29 | 0.26 |
| Luo 2019 | Serum miR‑146a | 64 | 12 | 14 | 68 | 3.26 | 0.43 |
| Wakefield 2004 | PDUS (≥ grade 2) | 34 | 5 | 14 | 17 | 2.11 | 0.60 |
| Hammer 2013 | PDUS | 98 | 15 | 22 | 65 | 2.96 | 0.37 |

Note: TP = true positive, FP = false positive, FN = false negative, TN = true negative.

lnDOR = ln((TP × TN) / (FP × FN)).

se(lnDOR) = √(1/TP + 1/FP + 1/FN + 1/TN).

All 2×2 counts are taken directly from Table 1 of the main manuscript.

The pooled lnDOR estimates in the network meta‑analysis were derived from these per‑study values using a random‑effects model.
